# Conventional and Algorithmic Music Listening before Radiotherapy Treatment: A Randomized Controlled Pilot Study

**DOI:** 10.3390/brainsci11121618

**Published:** 2021-12-08

**Authors:** Alfredo Raglio, Enrico Oddone, Ilaria Meaglia, Maria Cristina Monti, Marco Gnesi, Giulia Gontero, Chiara Imbriani, Giovanni Battista Ivaldi

**Affiliations:** 1 Istituti Clinici Scientifici Maugeri IRCCS, 27100 Pavia, Italy; ilaria.meaglia@icsmaugeri.it (I.M.); giulia.gontero@gmail.com (G.G.); chiara.imbriani@icsmaugeri.it (C.I.); giovannibattista.ivaldi@icsmaugeri.it (G.B.I.); 2 Department of Public Health, Experimental and Forensic Medicine, Section of Occupational Medicine, University of Pavia, 27100 Pavia, Italy; enrico.oddone@unipv.it; 3 Department of Public Health, Experimental and Forensic Medicine, Section of Biostatistics and Clinical Epidemiology, University of Pavia, 27100 Pavia, Italy; cristina.monti@unipv.it (M.C.M.); marco.gnesi@unipv.it (M.G.)

**Keywords:** music listening, music therapy, algorithmic music, breast cancer, radiotherapy, anxiety, stress

## Abstract

Music listening is a widespread approach in the field of music therapy. In this study, the effects of music listening on anxiety and stress in patients undergoing radiotherapy are investigated. Sixty patients with breast cancer who were candidates for postoperative curative radiotherapy were recruited and randomly assigned to three groups: Melomics-Health (MH) group (music listening algorithmically created, *n* = 20); individualized music listening (IML) group (playlist of preferred music, *n* = 20); no music group (*n* = 20). Music listening was administered for 15 min immediately before simulation and during the first five radiotherapy sessions. The State-Trait Anxiety Inventory (STAI) and the Psychological Distress Inventory (PDI) were administered before/after treatment. Cochran’s Q test and McNemar test for paired proportions were performed to evaluate if the proportion of subjects having an outcome score below the critical value by treatment and over time was different, and if there was a change in that proportion. The MH group improved in STAI and PDI. The IML group worsened in STAI at T1 and improved STAI-Trait at T2. The IML group worsened in PDI at T2. The No music group generally improved in STAI and PDI. Clinical and music listening-related implications are discussed defining possible research perspectives in this field.

## 1. Introduction

Music and music therapy are widely used as non-pharmacological modalities of intervention in different clinical and therapeutic settings of various nature. The literature includes a large number of studies organised according to scientific criteria as well as a large number of systematic reviews (including the Cochrane Reviews) that document the effects of those interventions [1,2,3,4,5]. Some of these reviews, along with single studies, relate to the use of music in hospital settings, in the pre-surgical and surgical phases [6], and to specific interventions in oncology [7], including radiation therapy/chemotherapy treatments [8,9,10,11,12]. In these contexts, application experiences are generally characterised by two factors: the first concerns the technique used, i.e., the use of music listening rather than active music therapy interventions (instrumental and/or vocal sonorous-music improvisation, but also, specific techniques such as song-writing and activities based on music-assisted relaxation); the second is concerned with the possible effectiveness of music on momentary symptoms, particularly anxiety, stress, and pain, resulting from disease conditions and/or particularly invasive medical procedures.

The present study includes the use of music listening linked to the concept of “Music Medicine” [6,13,14,15]. This is the possibility of modifying the psycho–physiological state of the patient through the targeted use of songs responding to specific musical parameters and structures that are able to interact with the psychosomatic imbalances to which the person is subjected in particular conditions, such as disease and surgery. Music, on the other hand, has also been shown to significantly influence vital parameters [16,17,18,19,20] and neurochemical processes [21,22,23], inducing the significant modifications of these aspects. Specifically in this randomised controlled study, in addition to proposing a traditional music listening approach, algorithmic music created for therapy [24,25,26,27,28,29] was used. This perspective represents an absolute innovation and creates considerable potential in the use of music listening for the treatment and health of the individual. The objective of the study was to verify if the perception of anxiety and stress in cancer patients undergoing radiotherapy is influenced by music listening. In particular, we investigated the effect of listening to music produced with the support of the Melomics-Health algorithm, compared to individualised music listening (listening to preferred music) and the absence of musical intervention.

## 2. Materials and Methods

### 2.1. Study Design and Sample Collection

Sixty Caucasian female patients with breast cancer and candidates for postoperative curative radiotherapy were recruited in the study of this randomised controlled trial with sequential enrollment. Patients were assigned to one of the following 3 groups: no music group (*n* = 20) to whom no musical listening was offered; individualized music listening group (IML group *n* = 20) who underwent a playlist of preferred music created with the collaboration of a music therapist; and a Melomics-Health group (MH group *n* = 20) who underwent a music listening algorithmically created in relation to the study objective. 

### 2.2. Inclusion and Exclusion Criteria 

Patients undergoing radiotherapy for the first time in their life were included. Patients with associated pathologies that preclude the understanding of the contents of the study (e.g., patients with psychiatric diseases and/or significant cognitive deficits), patients with significant hearing loss, patients that have previously undergone music therapy treatment based on music listening as well as patients with musical skills (musical training or practice ≥ 3 years) were excluded from the study. Additionally, patients who were candidates for partial hypo-fractionated breast irradiation lasting 10 sessions or less were excluded.

### 2.3. Randomisation

A randomisation list, according to a study design with parallel groups completely randomised (simple random allocation), was generated at the beginning of the study by a biostatistician by means of dedicated software and delivered to the principal investigator. In particular, in the randomisation list, the three treatment groups were distinguished by a non-informative label; the biostatistician provided the principal investigator with a list of all possible label-treatment combinations from which the principal investigator chose one; the chosen combination was made known to the biostatistician only at the end of the phase of statistical analysis of the collected data. Within the randomisation list, each subject was uniquely identified by a numerical code that allowed its identification throughout the duration of the study. Patient identification data was maintained in a separate and adequately protected database.

### 2.4. Treatments 

The MH group listened to music produced by Melomics-Health (15 min, 5 songs of about 3 min each). Melomics-Health technology was born from the idea of creating musical pieces with a therapeutic purpose rather than aesthetic purpose. The music produced by this algorithm is reduced to essential parameters so as to be characterized by a limited number of music variables (mainly related to tempo, time signature, timbre, pitch range, rhythm, key signature, dynamics, and duration). The algorithm allows a possible manipulation of these variables, relating the music structure with its possible effects. Each bar, as well as the general music design, can be modified by changing predefined music parameters in relation to their effectiveness. These features make the algorithmic music flexible and able to be modulated, facilitating its use in the therapeutic field. On this basis of some “music models” aimed at relaxation/de-activation, activation and distraction have been created, considering these aspects as important factors in the therapeutic contexts.

In this study the music proposed (based on relaxing/de-activating music model) was designed to reduce the state of anxiety and stress given by radiation therapy. The musical content was designed to act as a psychological de-activator and to be relaxing. All parameters and structures maintained low levels and a consistent course. The music was based on a regular pattern, a monody with a reduced musical density; the time was unchanged and there were no significant dynamic and tonal variations. 

Similarly, the IML group listened in the same way during the phases of the intervention using a playlist of songs that are deemed relaxing and emotionally meaningful for the patients. This playlist lasting approximately 15 min was built by the patients and the music therapist in the days prior to the experiment. The patients assigned to this group also listened to the music using earphones. The no-music group did not perform any type of musical listening. Patients were asked to arrive about 20 min in advance of the scheduled treatment time, to begin the listening experience 15 min before the simulation and the first 5 radiotherapy sessions. The listening experience took place in a quiet room inside the radiotherapy service where the patients were alone. Patients listened to the music using earphones in order to focus their attention on the sound-musical component and to isolate themselves from the context.

### 2.5. Assessment

Patients underwent a psychological assessment that focused on the dimensions of anxiety and distress related to the planned medical procedure and more generally to the clinical condition. At the time of the enrolment of patients (screening), a clinical/anamnestic assessment was performed, the presence of inclusion criteria was verified and eligible patients who adhered to the study were asked to sign the informed consent. At baseline (T0), at the end of treatments (T1) and at follow-up (T2, 2 weeks after the fifth session of radiotherapy) the State-Trait Anxiety Inventory (Form Y, STAI) [30] and the Psychological Distress Inventory (PDI) [31] were administered. 

The STAI-S of the State-Trait Anxiety Inventory comprises 20 items. Items are answered on a 4-point Likert scale ranging from 1 (*not at all*) to 4 (*very much so*), with the total score ranging from 20 to 80. Higher scores indicate increased levels of anxiety symptoms. A cutoff score of 40 is commonly used to define clinical levels of anxiety.

The PDI is a 13-item self-administered questionnaire aimed at measuring psychological distress in cancer patients. It has been originally developed and validated in an Italian context. The respondents are asked to choose the response option that best describes how they have felt in the past week for each item. It has been shown that a score over 35 is indicative of clinically significant distress.

Additionally, at the end of the study, MH and IML groups were also provided with a questionnaire regarding music listening. On the basis of a Visual Analogue Scale (VAS, scores 0–10), the participants were asked to indicate their enjoyment of the music, their level of evocation of the images/emotions and their desire to listen to the proposed pieces again. Participants were also asked to describe their emotions while listening to music in the questionnaire. The prevailing emotion during the music listening experience has been chosen from a given list, but also with the possibility to indicate in a free space an emotion not included in that list. The reported emotions were subsequently classified by the researchers as positive, negative, or neutral. 

Psychological evaluations, randomisation and statistical processing of the data were blinded. To reduce the possibility of bias in the study, the evaluators did not know the allocation of patients in the different groups of study, and the statisticians processed the anonymised data. 

### 2.6. Statistical Analysis

All variables collected by the interviewers were summarised as median (interquartile range) or percentage frequencies by group of treatment. The Kruskal–Wallis test was used to compare age among groups of treatment at baseline. 

In order to evaluate if the proportion of subjects with an outcome score below the critical value by treatment and over time were different, a Cochran’s Q test was performed globally. To evaluate if there was a change in proportion of subjects with an outcome score below the critical value between each pair of time phases by treatment, the McNemar test for paired proportion was used. The chi-square test or Fisher exact test were used to compare, by treatment, the proportion of subjects with an outcome score below the critical value for each time phase of the study (T1, T2, T3). The data were analysed using the software Stata 15 (2017, College Station, TX 77845, USA).

## 3. Results

**Figure 1:**
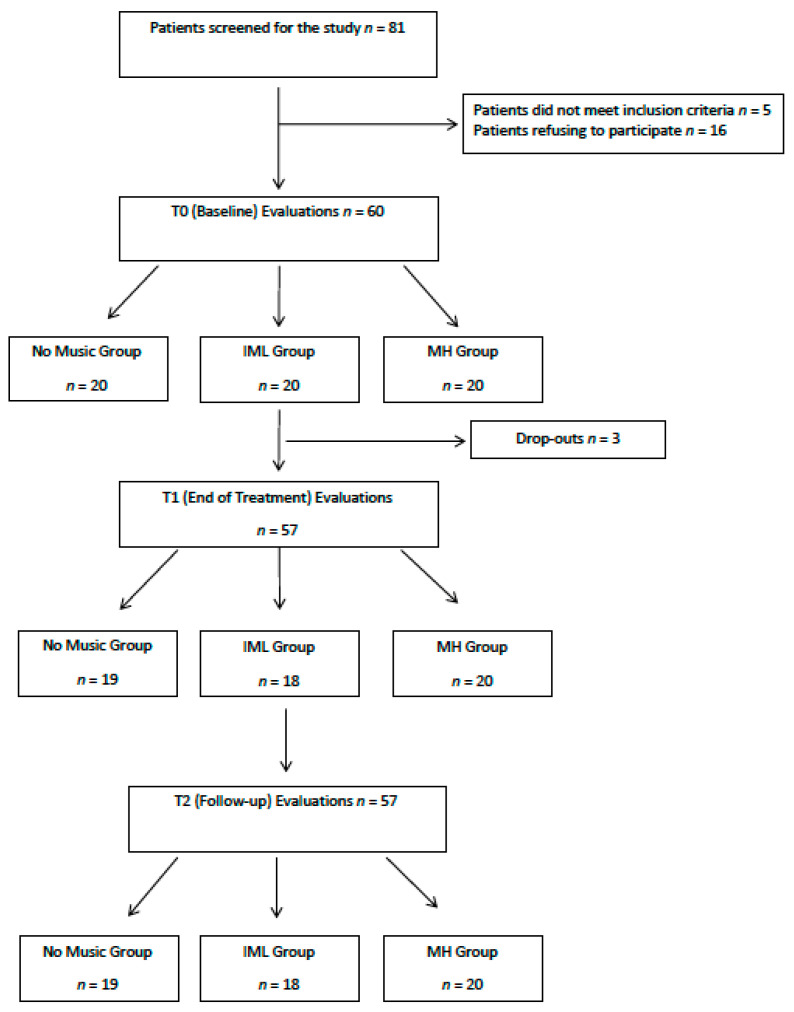
Distribution of patients and drop-outs in no music group, individualised music listening group (IML) and Melomics-Health group (MH) at screening, baseline (T0), end of treatment (T1) and follow-up (T2).

The flowchart reported in Figure 1 summarises the steps of the study.

**Figure 2:**
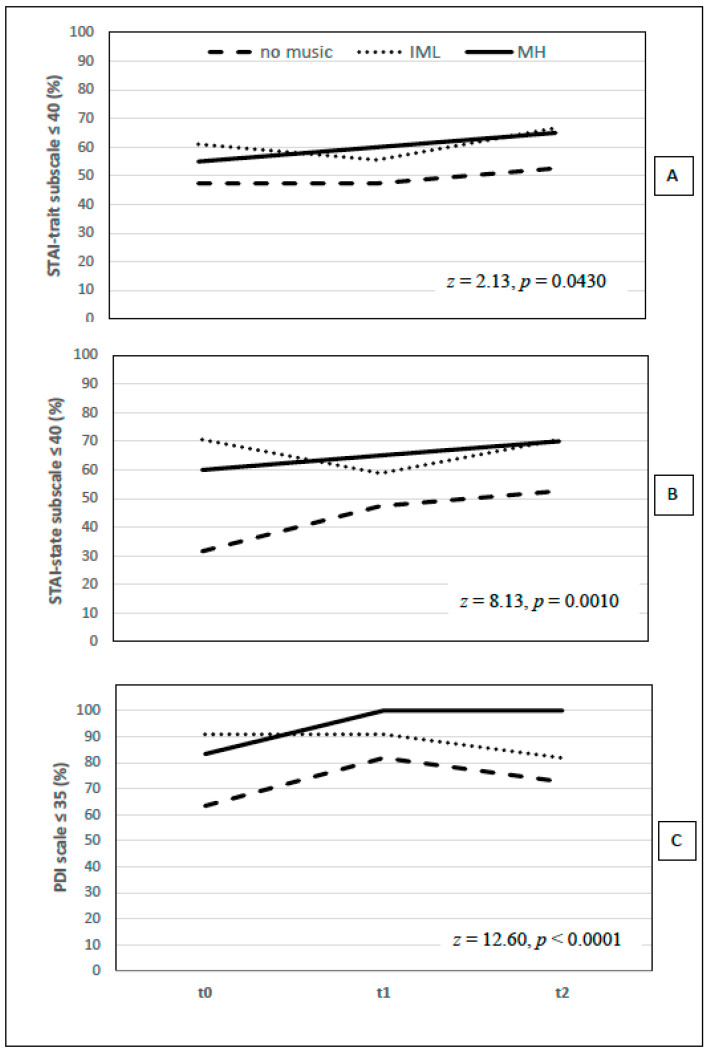
Proportion of patients with a State-Trait Anxiety Inventory (STAI, **A**,**B**) or Psychological Distress Inventory (PDI, **C**) scores below the critical value at baseline (t0), end of treatment (t1) and at follow-up (t2). Cochran’s Q test was used to evaluate if the proportion of patients having an anxiety score below the critical value for the STAI sub-scales and PDI scale changed over time, depending on the treatment. A *p*-value ≤ 0.05 was considered statistically significant.

Patients’ age distribution was similar among groups (median and range were, respectively, for the no music group: 59, 51–68; for the IML group: 54.5, 48–68 and for the MH group: 58, 55–67). Main results of the study are summarised in Figure 2. Overall, the proportion of subjects with an outcome score below the critical value by treatment and over time were different (*p* = 0.0430 for STAI-Trait, *p* = 0.0010 for STAI-State and *p* < 0.0001 for PDI scales). 

STAI-Trait scores showed an improvement in the trend of MH group at T1 and T2 (T1 vs. T0: +5%; T2 vs. T1: +5%). In the no music group, scores did not change at T1 and slightly improved at T2 (T2 vs. T1: +5.26%) and in the IML Group, scores worsened at T1 (T1 vs. T0: −5.55%) and improved at follow-up (T2 vs. T1: +11.11%).

Additionally, the trend of the STAI-State scores showed an improvement in MH and no music groups at T1 (T1 vs. T0: MH group +5% and no music group +15.79%). This improvement continued at T2 (T2 vs. T1: MH group +5% and no music group +5.26). On the contrary, the IML group worsened at T1 (T1 vs. T0: −11.77%) and improved at follow-up (T2 vs. T1: +11.77%).

PDI scores showed an improvement in the trend of MH and no music groups at T1 (T1 vs. T0: MH group = +16.67%; no music group = +18.42%). At follow-up, scores worsened in the no music group (T2 vs. T1: −9.12) and remained unchanged in the MH group. Moreover, in the IML group, the scores remained unchanged at T1 and worsened at follow-up (T2 vs. T1: −9.08%). 

The levels of enjoyment of the music and the evocation of images/emotions (median of the two VAS scores, eight in both groups) did not differ significantly between the two groups of people exposed to musical listening regarding listening to the proposed songs again (median of VAS scores: IML group = 9.0; MH group = 8.5). Both groups also reported a clear prevalence of positive emotions (IML = 94.74% and MH = 89.47%).

## 4. Discussion

The study does not show clear results comparing musical listening and no music. This could be derived from methodological components, such as the choice of the time of listening (immediately before radiotherapy) and, also, from the reduced time of exposure to the stimulus (15 min). In addition, the different levels of severity at baseline (IML group with more marked severity than the MH or no music groups). A large number of patients declined to participate in the study. In this sense, the intervention may have occurred during a particularly difficult psychological moment or at a time when patients had low interest in non-pharmacological interventions, as well as reluctance to commit to the time required for listening to music before the radiotherapy session. However, some considerations are of particular interest, for example, in the comparison between the two types of musical stimuli. Algorithmic music listening (MH group) in fact, compared to listening to conventional music (preferred music, IML group), showed a positive trend in the reduction of anxiety and stress. This suggests that, with regard to specific symptoms such as anxiety and stress, the structure and musical parameters constitute elements of particular relevance (with respect to subjective factors related to the musical preferences of the subjects) in determining the effects of music. Another finding of particular interest that emerged from the questionnaire data is the subjective impact of algorithmic music on the participants. As with conventional self-selected music, this type of music seems evocative and pleasing, arousing substantially positive emotions in the same manner.

Therefore, algorithmic music can be considered a promising therapeutic tool comparable to conventional music [27]. This type of music can be modelled and adapted to the person and his/her therapeutic needs in other clinical contexts (e.g., in the context of chronic and acute pain, behavioural disturbances, and sleep medicine). Algorithmic music, because it is more detached from cultural factors and emotional memories, can also be a promising stimulus for neuroscientific research. Through the influence of algorithmic music sound structures and musical parameters, the cultural dimension of music is in part bypassed. The Melomics-Health algorithm should be considered as a work in progress: in fact, the effectiveness of this approach requires further confirmation. For this reason, the development of this technology includes studies concerning clinical aspects [25,26,27] but also studies aimed at understanding the physiological effects of this type of music [32]. The goal of these experiments is to understand more deeply the effects of musical structures and features by finalizing their use for therapeutic purposes. Considering the use of music listening approaches in radiotherapy, future studies with larger samples should take into account a greater exposure to the sonorous stimuli, such as the addition of music listening some days before and during radiotherapy.

## Data Availability

Main data generated or analysed during this study are included in this article. Further enquiries can be directed to the corresponding author.
